# Challenge of a dual burden in rapidly aging Delaware: Comorbid chronic conditions and subjective cognitive decline

**DOI:** 10.1371/journal.pgph.0000579

**Published:** 2022-08-25

**Authors:** Sangeeta Gupta

**Affiliations:** Department of Public and Allied Health Sciences Delaware State University, Dover, Delaware, United States of America; Instituto Nacional de Geriatria, MEXICO

## Abstract

**Background:**

Epidemiologic trends forecast a “dual burden”- increase in both physical chronic diseases and Alzheimer’s disease (AD)- for Delaware. Estimating the burden and characteristics of this “dual burden” is critical. Cognizant of the unavailability of precise models to measure AD, SCD—a population-based measure- was used as an alternative. The primary objective was to delineate selected chronic conditions among Delaware adults with SCD in order to present: (i) prevalence of SCD by select sociodemographic characteristics, (ii) compare the prevalence of chronic conditions among people with and without SCD, and (iii) compare the prevalence of SCD associated functional limitations in Delawareans with and without comorbid chronic conditions.

**Methods:**

Combined data (2016 and 2020) for Delaware were obtained from the Behavioral Risk Factor Surveillance System. Analyses included 4,897 respondents aged 45 years or older who answered the SCD screening question as “yes” (n = 430) or “no” (n = 4,467). Descriptive statistics examined sociodemographic characteristics and chronic conditions in Delawareans with and without SCD.

**Results:**

Overall, 8.4% (CI: 7.4–9.5) of Delaware adults reported SCD. Delawareans with SCD were more likely to be in the younger age group (45–54 years), less educated, low income and living alone. Over 68 percent had not discussed cognitive decline with a health care professional. More than three in four Delawareans with SCD had a 1.5 times higher prevalence of having any one of the nine select chronic conditions as compared to those without SCD. Adults with SCD and at least one comorbid chronic condition were more likely to report SCD-related functional limitations.

**Conclusions:**

Delaware cannot afford to postpone public policies to address the dual burden of SCD and chronic conditions. Results from this study can help public health stakeholders in Delaware to be informed and prepared for the challenges associated with cognitive decline and comorbidity.

## Background

Subjective cognitive decline (SCD) associated with aging is characterized by self-experience of decline in cognitive performance and may preclude onset of Alzheimer’s disease (AD) [1,2]. Delaware is one of the states aging fastest in the United States [2]. Major epidemiologic trends forecast a “dual burden”- increase in both physical chronic diseases and AD- for Delaware [3–6]. The confluence of cognitive impairment due to AD and comorbid chronic conditions, complicate access to health care, interfere with self‐management, and necessitate reliance on caregiver resources [1,5].

The proportion of Delaware’s population that is 60 and older is growing more rapidly than other population categories [3,4]. Delaware’s Division for Aging estimates a 50.4 percent increase in Delaware’s 60 and older population by the year 2030, from 2015 baseline level [3]. Aging societies brings significant health care challenges in the form of a higher risk of physical chronic diseases such as diabetes, heart disease, cancer, chronic lower respiratory diseases, and stroke [4,5]. Delaware’s carries an extremely high burden of multiple chronic conditions, up to 38.5% [6]. 2020 American Health Rankings report recognizes this as a major health challenge [7]. Even more disturbing is the fact these figures are an underestimate of the actual burden, since these do not include AD [8]. Between 2020 and 2025, the number of persons aged 65 and over with AD in Delaware is expected to grow by 21.1% (from 19,000 people to 23,000 people) [9]. AD is a chronic neurodegenerative disease [8,9]. Unlike other chronic diseases, few or no effective treatments are available for AD. Hall mark feature of AD—progression in an irreversible manner- is responsible for large socioeconomic and personal costs [9]. Early detection and intervention may slow disease progression and provide an opportunity to caregivers and family for future plans [9].

Cognitive impairment along with comorbid physical chronic conditions puts Delawareans at a higher risk of poor health outcomes and high care costs, particularly regarding chronic disease self- management [3,9]. Estimating the burden and characteristics of the Delaware population affected by this “dual burden” is important for the Delaware public health community and policymakers in planning targeted interventions.

A population-based study to estimate the prevalence of AD and comorbid chronic conditions would be ideal. Unfortunately, population -based estimates of AD are based on complex models prone to imprecision [10–12]. However, there is an alternative option. SCD can be easily measured at a population level and thus provides an opportunity for estimating the extent and burden of cognitive issues. SCD can be one of the earliest signs of AD but comes with a caveat [13]. Not everyone with SCD will develop AD. It has been postulated that as many as 60% reporting SCD will deteriorate to a diagnosis of AD over a 15-year period [13,14]. Irrespective of its predictive value for AD, SCD will impact a person’s ability for self-care, activities of daily living and managing comorbid chronic conditions [15].

In the United States, a population based SCD measure exists through the Centers for disease Control and Prevention (CDC) Behavioral Risk Factor Surveillance System (BRFSS) [16]. Delaware administered the optional SCD module through BRFSS in 2016 and most recently, in 2020. To the best of our knowledge, no population-based studies have explored and done a detailed characterization of this “dual burden”- SCD and comorbid chronic conditions -in Delaware. This study aims to delineate comorbid chronic conditions among Delaware adults with SCD in order to present: (i) prevalence of SCD by select sociodemographic characteristics including social determinants of health (SDOH) such as education, living alone, income and health care, (ii) compare the prevalence of chronic conditions among people with and without SCD, and (iii) compare the prevalence of SCD associated functional limitations in Delawareans with and without comorbid chronic conditions. It is important to note here that this study findings will come with a caveat. AD is not the only cause of subjective cognitive decline and various other conditions can be associated with subjective memory complaints, such as psychiatric disorders (depression, anxiety) or normal aging. Prior research has revealed an important correlation between SCD and mood disorders (such as depression) [16,17]. It has been argued that subjective decline in cognition reflects affective symptoms (i.e., depression and anxiety) rather than actual cognitive issues.

Living with significant cognitive impairment and co-occurring chronic conditions is an important issue for public health in aging society. Bolstering SCD research efforts will enable improved characterization of this vulnerable Delaware population, support health care and other providers in coordinating and managing care to meet the needs of this fast -increasing demographic. Our hypothesis is that Delawareans with SCD have a significantly stronger association with physical chronic conditions, face more functional limitations and are more vulnerable in terms of the SDOH.

## Materials and methods

### Data source

Behavioral Risk Factor Surveillance System (BRFSS), established in 1984, is the largest random- digit- dialed telephone health survey of noninstitutionalized U.S. adults aged 18 years or older, conducted annually by the Centers for Disease Control and Prevention (CDC) in all 50 states, the District of Columbia, and US territories [18]. In 2015, BRFSS added a six-question optional module on cognitive decline administered to adults 45 years and older. Delaware administered the SCD module in 2016 and 2020.

**Fig 1:**
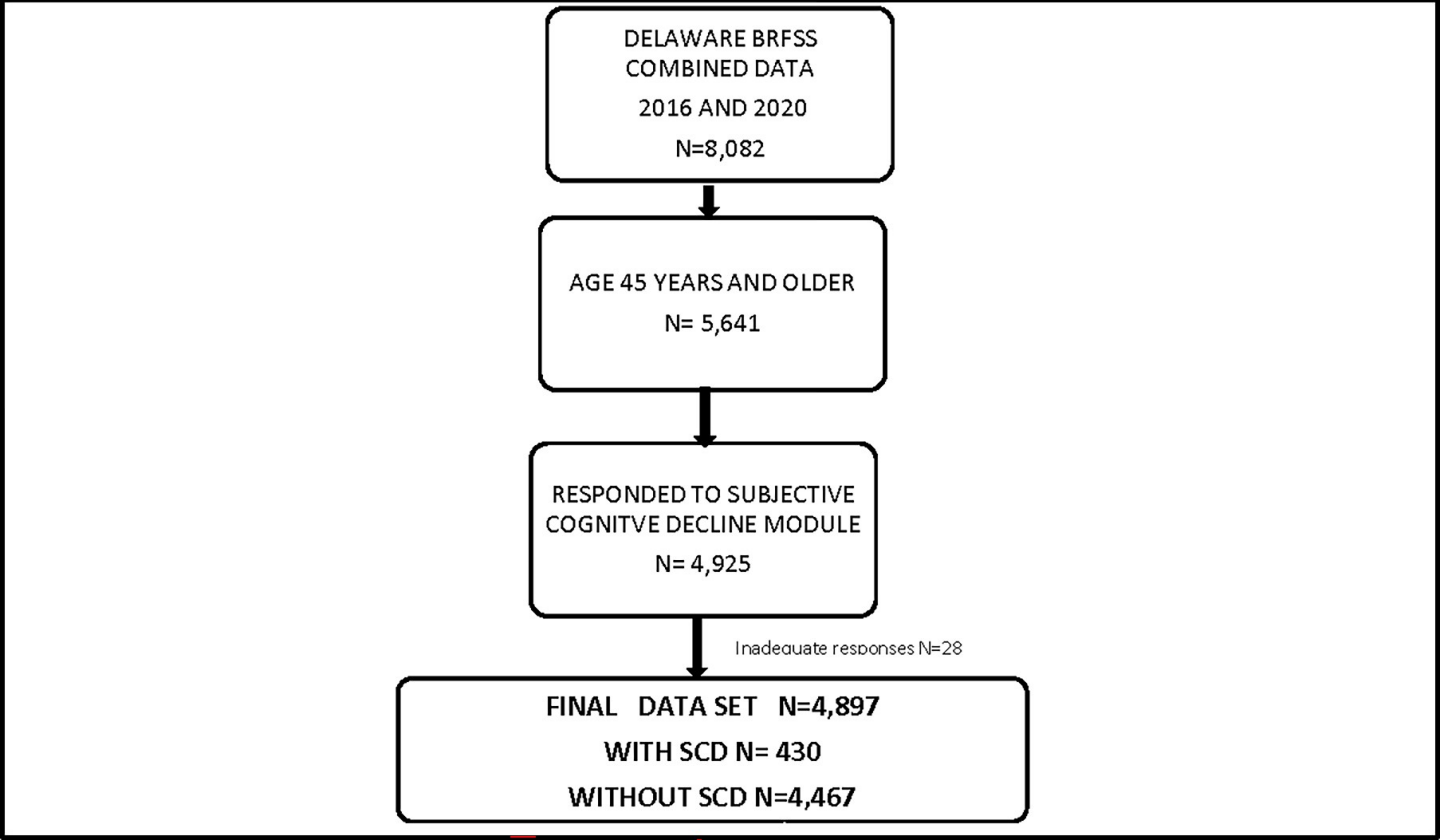
Sampling technique.

Combined Delaware BRFSS data for 2016 and 2020 (n = 8,082) were analyzed for this study. 5,641 respondents were aged 45 years or older. In the 45 years or older data set, 4,925 Delawareans responded to the cognitive decline module. Excluding inadequate responses (n = 28), final data set (n = 4,897) included 430 respondents with SCD and 4,467 without SCD (Fig 1). The excluded respondents were similar to those included in the study in terms of age group and race/ethnicity. Delaware sample was weighted at the state level and analyses conducted with BRFSS specified complex sampling procedures to appropriately stratify and weigh the data [19].

Delaware BRFSS response rates were 47.5% and 44.7% (landline), 38.1% and 35.5% (cell phone) and 43% and 38.5% (combined landline and cell phone) in 2016 and 2020 respectively. Response rates for BRFSS are calculated using standards set by the American Association for Public Opinion Research (AAPOR) Response Rate Formula #4 [20]. The response rate is the number of respondents who completed the survey as a proportion of all eligible and likely-eligible people. For detailed information please see the BRFSS Summary Data Quality Report [21].

### Variables

#### Outcome variable

*Subjective cognitive decline*. Delawareans who answered “yes” to the question, “During the past 12 months, have you experienced confusion or memory loss that is happening more often or is getting worse?” were included as subjects with SCD. They were then asked four subsequent questions that assessed SCD attributed impairment in activities: (i) how often SCD caused them to give up day-to-day activities such as cooking, cleaning, taking medications, driving, or paying bills; (ii) how often they needed assistance with these day-to-day activities; (iii) how often were they able to get the help they needed; and (iv) how often did SCD interfere with their ability to work, volunteer, or engage in social activities about the home. Reply as “always, usually, or sometimes” was considered as an affirmation of SCD related functional limitation. In addition, a fifth question asked if they had talked to a health-care professional about their confusion or memory loss.

#### Independent variables

Data of 4,897 Delawareans were categorized by age group (45–54;55–64 and ≥65 years), sex (male and female) and race (i) White—Non-Hispanic; ii) Black-Non-Hispanic and iii) Hispanic).

Social determinants of health were characterized by the following variables: 1)educational attainment (did not graduate high school; graduated high school or equivalent; attended college or technical school; and graduated from college or technical school); 2)lives alone or does not live alone; 3) yearly income from all sources (<15,000; 15,000-<25,000;25,000-<35,000; 35,000-<50,000; and ≥ 50,000); and 4) primary health insurance was determined as any kind of health care coverage, including prepaid plans such as health maintenance organizations (HMOs), or government plans such as Medicare, or Indian Health Service or none.

Nine chronic diseases and two health behaviors were assessed for comorbid status with SCD. Participants were identified as having a diagnosed chronic disease if they had been told by a doctor or other health care provider that they ever had (i) angina; (ii) arthritis; (iii) asthma; (iv) cancer (other than skin cancer); (v) chronic kidney disease; (vi) chronic obstructive pulmonary disease; (vii) diabetes (not including gestational, borderline, or prediabetes); (viii) heart attack; and (ix) stroke. Physical exercise was assessed by the following question: “During the past month, other than your regular job, did you participate in any physical activities or exercises such as running, calisthenics, golf, gardening, or walking for exercise?”. Delawareans smoking status was assessed as “current”, “past” or “never” through a BRFSS calculated variable. Reporting of chronic conditions through BRFSS comes with a limitation. As BRFSS collects information only on conditions confirmed by a doctor or health professional, there is a potential for underreporting of conditions that were undiagnosed or were not recalled by the respondent during the telephone interview.

Delawareans with SCD were compared with Delawareans without SCD for association with selected chronic conditions and SDOH such as education, income, living alone and health care coverage. Prevalence of functional limitations in Delaware adults with SCD was further assessed by comorbid chronic condition status.

### Statistical analyses

Disparities amongst Delawareans by SCD status were assessed by calculating weighted estimates accounting for the complex BRFSS sampling methodology. Statistical differences were determined with Rao–Scott chi-square tests. Prevalence ratios (PR) with 95% confidence intervals (CIs) were calculated as unadjusted values. All analyses were performed by using appropriate survey commands in SAS 9.4 (SAS Institute Inc., Cary, NC) was used for all analyses. Statistical significance was denoted by a P value of < .05. As per standard BRFSS data suppression guidelines, if the confidence interval was more than 20 points wide, results for that cell were suppressed.

## Results

### Select characteristics of delware adults with SCD (Table 1)

8.4% (95% CI: 7.4–9.5) of Delawareans aged 45 years or older reported SCD.

Proportion of females with SCD was higher than males with SCD among Delaware adults. However, differences by gender were not significant (p = 0.2520). SCD prevalence increased with age group being highest in the 65 and older age group, 48.5% (95% CI: 42.1–54.8) although nor statistically significant (p = 0.1126). Across groups defined by race and ethnicity, higher percentages of Delaware adults with SCD were Whites, 78.9% (95%CI: (73.6–84.2) compared to Blacks and Hispanics (p = 0.5515) (Table 1).

**Table 1:** Demographic Characteristics of Delaware Adults Aged 45 Years or Older by Subjective Cognitive Decline (SCD) Status, Behavioral Risk Factor Surveillance System (Combined 2016 and 2020).

N = 4,897		
	With Subjective Cognitive Decline	Without Subjective Cognitive Decline
	Overall	Overall
	n = 430	n = 4,467
	% (95% CI)	% (95% CI)
**Overall** ^	8.4 (7.4–9.5)	91.6(90.5–92.6)
**Sex** *		
Male	39.1(29.3–48.7)	45.1(42.1–47.9)
Female	60.9(51.3–70.7)	54.9(52.1–57.9)
**Age (y)** *		
45–54	26.3(20.5–32.1)	27.1(25.3–28.9)
55–64	25.2(19.9–30.6)	30.8(29.1–32.6)
≥65	48.5(42.1–54.8)	42.1(40.2–43.9)
**Education ^**		
< High School	17.3(11.9–22.7)	8.4(7.2–9.7)
High School	38.5(32.4–44.8)	31.3(29.4–33.1)
Attended College	24.1(18.7–29.5)	29.4(27.6–31.3)
Graduated College	19.9(15.4–24.6)	30.9(29.2–32.5)
**Income ($)** ^		
<15000	16.8(11.9–21.6)	7.2(6.1–8.4)
15,000—< 25,000	29.1 (22.7–35.4)	13.9 (12.5–15.4)
25,000 - <35,000	10.9(6.8–14.9)	10.2(8.8–11.5)
35,000 - <50,000	14.6 (10.1–19.2)	13.3(11.9–14.7)
≥ 50,000	28.7(21.9–35.4)	55.4(53.3–57.5)
**Living alone** *		
Yes	31.4(23.1–39.6)	27.5(25.1–29.9)
**Health Care** *		
No	4.1(1.7–6.5)	4.8 (3.9–5.7)
**Race** *		
White,non-Hispanic	78.9(73.6–84.2)	76.5 (74.6–78.5)
Black,nonHispanic	17.5(12.4–22.6)	20.2(18.3–22.1)
Hispanic	3.7(1.7–5.6)	3.3(2.7–3.8)

*Note*: frequencies presented are unweighted. Percentages and confidence intervals are weighted based on state population sizes.

*not significant compared to group without SCD. ^ ***significant*** compared to group without SCD.

Amongst SDOH, significant differences were observed by educational status and annual income. SCD prevalence was significantly lower among college graduates (p < .0001). Nearly 60% of Delaware adults with SCD had high school or less than high school education.

SCD prevalence was significantly higher among low income groups (p < .0001). Over seventy percent of Delaware adults with SCD annual household income from all sources was less than $50,000 (Table 1).

Of Delaware adults with SCD, 31.4% (95% CI: 23.1–39.6) reported living alone. 4.1% (95% CI: 1.7–6.5) of Delawareans with SCD were uninsured. (Table 1).

### Comorbid chronic conditions among Delaware adults with SCD (Table 2)

In Delawareans with SCD there was a significantly higher prevalence of all nine selected chronic conditions compared to those without SCD (Table 2). More than three in four Delawareans with SCD had a 1.5 times higher prevalence of having any one of the nine select chronic conditions as compared to those without SCD (*p* < .0001).

**Table 2:** Delaware Adults Aged 45 Years or Older Who Reported Comorbid Chronic Diseases/Cognitive Risk factor by Subjective Cognitive Decline (SCD) Status, Behavioral Risk Factor Surveillance System (BRFSS) (Combined 2016 and 2020).

**At least one chronic condition**		**% (95% Confidence Interval)**				
With SCD				76.7(71.4–81.9)		
Without SCD				51.7 (49.7–53.6)		
Prevalence Ratio				1.5(1.4–1.6) ^a^		
**Selected Chronic Condition**		**% (95% Confidence Interval)**				
**Angina** #						
With SCD		17.2(11.8–22.7)				
Without SCD		6.6(5.7–7.5)				
Prevalence Ratio		2.6 (1.9–3.7) ^a^				
**Heart Attack** #						
With SCD		18.4(12.8–23.9)				
Without SCD		7.4 (6.4–8.4)				
Prevalence Ratio		2.5(1.8–3.5) ^a^				
**Stroke** #						
With SCD		13.7(9.5–17.8)				
Without SCD		4.3(3.5–5.1)				
Prevalence Ratio		3.2(2.3–4.6) ^a^				
**Diabetes** #						
With SCD		27.4 (21.4–33.4)				
Without SCD		18.2 (16.6–19.8)				
Prevalence Ratio		1.5(1.2–1.9) ^b^				
**Asthma**						
With SCD		20.4(15.5–25.2)				
Without SCD		11.7(10.3–12.9)				
Prevalence Ratio		1.7(1.3–2.3) ^a^				
**Cancer**						
With SCD		18.8 (13.2–24.4)				
Without SCD		11.4(10.2–12.5)				
Prevalence Ratio		1.7(1.2–2.3) ^c^				
**Chronic Kidney Disease**						
With SCD		8.4 (5.4–11.5)				
Without SCD		5.1(4.1–5.9)				
Prevalence Ratio		1.7(1.1–2.5) ^d^				
**Arthritis**						
With SCD		60.9(51.7–70.1)				
Without SCD		38.5(35.6–41.4)				
Prevalence Ratio		1.6(1.3–1.9) ^a^				
**Chronic Obstructive Pulmonary Disease**						
With SCD		22.5 (17.5–27.6)				
Without SCD		8.3(7.3–9.3)				
Prevalence Ratio		2.7(2.1–3.5) ^a^				
**Lack of exercise**						
With SCD		47.5(41.1–53.8)				
Without SCD		27.5 (25.7–29.3)				
Prevalence Ratio		1.7(1.5–2.1) ^a^				
**Smoking**						
With SCD		59.4(53.1–65.7)				
Without SCD		47.7(45.7–49.6)				
Prevalence Ratio		1.2(1.1–1.4) ^e^				

# cognitive risk factors—angina, heart attack, stroke, and diabetes.

a p < .0001

b p = 0.001

c p = 0.0023

d p = 0.0103

e p = 0.0006.

The three cognitive risk factors (angina, heart attack, and stroke) were top ranking in prevalence amongst Delaware adults with SCD, up to three times higher as compared to adults without SCD (*p* < .0001). Prevalence of stroke was the highest (PR = 3.2, 95% CI: 2.3–4.6) followed by angina (PR = 2.6, 95% CI: 1.9–3.7) and heart attack (PR = 2.5, 95% CI: 1.8–3.5).

Prevalence of other chronic conditions too were significantly higher in Delawareans with SCD. Prevalence of diabetes (PR = 1.5, 95% CI: 1.2–1.9), arthritis (PR = 1.6, 95% CI: 1.3–1.9), asthma (PR = 1.7, 95%CI: 1.3–2.3), cancer (PR = 1.7,95% CI: 1.2–2.3), and chronic kidney disease (PR = 1.7, 95% CI: 1.1–2.5) were nearly two times higher among Delaware adults with SCD compared to the non SCD group. Prevalence of chronic obstructive pulmonary disease was nearly three times more in Delawareans with SCD than without SCD (PR = 2.7,95%CI: 2.1–3.5).

Lack of physical activity and smoking were significantly higher among Delawareans with SCD. Adults with SCD were nearly two times less likely to exercise (PR = 1.7, 95% CI: 1.5–2.1) than Delaware adults without SCD. The SCD subgroup of Delaware adults were also more likely to smoke (PR = 1.2, 95% CI: 1.1–1.4).

### Functional limitations among DELWARE adults with SCD by chronic condition status (Table 3)

Delaware adults with SCD and at least one comorbid chronic condition were more likely to report SCD-related functional limitations compared to those with SCD and no reported comorbid chronic conditions (p < .0001) (Table 3).

**Table 3:** SCD-related functional limitation in Delaware Adults aged ≥45 years with subjective cognitive decline (SCD), by comorbid chronic condition status in preceding 12 months—Behavioral Risk Factor Surveillance System (combined data 2016 and 2020).

	At least one comorbid chronic condition	No comorbid chronic condition
	**% (95% Confidence Interval)**	**% (95% Confidence Interval)**
**Given up day-to-day chores due to confusion or memory loss** ^a^		
Yes	37.6 (30.3–44.9)	23.3 (13.1–31.1)
**Need assistance with day-to_day activities due to confusion or memory loss** ^b^		
Yes	39.9 (32.5–47.2)	24.7 (13.2–32.5)
**Confusion or memory loss interfere with work or social activities** ^c^		
Yes	34.8 (27.5–42.1)	28.7 (18.7–37.8)
**Discussed your confusion or memory loss with a health care professional** ^d^		
No			48.7 (41.3–56.2)	68.1 (56.2–80.1)

^a^ p = 0.0333

^b^ p = 0.0409

^c^ p = 0.4132

^d^ p = 0.0102

Less than half (48.7%) of Delaware adults with SCD and at least one comorbid chronic condition reported not discussing their more frequent of worsening confusion or memory loss with a health care professional (p = 0.0102). This percentage was significantly higher than for those without comorbid chronic conditions, where 68.1%(95% CI: 56.2–80.1) of adults did not discuss SCD with a health care professional.

Those with at least one comorbid chronic condition were more likely to report having to always, usually, or sometimes give up household activities because of SCD when compared to those with SCD but with no comorbid chronic conditions (p = 0.033).

Nearly four in ten Delawareans with SCD always, usually, or sometimes needed assistance with day-to -day activities due to confusion or memory loss compared to one in four Delawareans without SCD (p = 0.0409).

Greater number (34.8%) of Delaware adults with SCD reported confusion or memory loss (always, usually, or sometimes) to interfere with work or social activities as compared to Delaware adults without SCD (24.7%), although the results were not statistically significant.

## Discussion

Nearly one in 12 Delaware adults aged 45 years or older reported SCD. Burden of SCD in Delawareans was further compounded by comorbid chronic conditions. Compared with those without SCD, Delawareans aged 45 years and older with SCD were two or three times more likely to have angina, heart attack, stroke, diabetes, asthma, COPD, cancer, arthritis, or kidney disease. Close to seventy seven percent of Delawareans with SCD have at least one other chronic health condition. This study adds state level presentation of similar national level associations through prior research [9,20,22].

Comorbid chronic conditions can complicate health issues in more than one respect. First and foremost, chronic conditions can increase the risk of cognitive decline and progression to AD [9,20]. Microvascular damage caused by these “cognitive risk factors” can result in a gradual but often more global decline in cognitive function [23]. The second, equally important concern relates to management of comorbid conditions in the presence of SCD. Because of impaired cognitive function, medication instructions or nutritional / physical activity regimes provided by clinicians becomes a challenge. Thus, self-management–a key notion for persons with chronic illness–does not apply to a person with SCD [9,23]. Poorly managed chronic diseases could lead to further cognitive impairment [20,24,25].

Delawareans with SCD were also found to have a significantly higher association with both lack of exercise and smoking tobacco. Study results document smokers are likely to experience faster 10-year cognitive decline in global cognition and executive function [26,27]. Physical activity protects the heart and may also protect the brain and reduce the risk of developing AD [28–30]. The 2020 Lancet Commission on dementia prevention clearly recognizes the role of these modifiable risk factors and states that addressing these might prevent or delay up to 40% of dementia cases [31]. In highlighting these associations, this study provides an opportunity for Delaware policy makers to address modifiable risk factors that may delay cognitive decline in Delawareans.

Differences in SDOH—conditions in places where people are born, live, learn, work, and play- can have a profound effect on a person’s health, including risk for AD [32]. Study findings demonstrated a significantly higher burden of adverse determinants such as low income and less education. Proportion of Delawareans experiencing SCD was lower among college graduates and those with higher incomes. Researchers believe having more years of education builds up “cognitive reserve (coping ability of the brain to improvise and execute) [32,33].

It is interesting to note that more than fifty percent of Delawareans with SCD were less than 65 years of age. More disturbing was the finding that more than one in four Delawareans with SCD were in the younger age group of 45–54 years. These finding forecasts important health and economic impacts for Delaware. Adults in the age group between 45–54 years are largely considered to be: i) in their heyday of productive work and income, ii) at a time to contribute to maximum to their retirements, and iii) serve as revenue driving consumers [33–35]. Delawareans may have to leave workforce entirely due to SCD or reduce work time. In either case there would be dire financial consequences for these adults and their families.

Unlike previous national studies [22,33,36–38] on SCD, no significant disparities by race were observed among Delawareans with and without SCD. Of Delawareans with SCD, nearly eighty percent were Whites followed by Blacks (17.5%) and Hispanics (3.7%).

Amongst Delawareans with SCD, more than 60 percent were females. Although not statistically significant, this is a finding of interest. More women- almost two-thirds—than men have AD [9,39]. This difference is most likely attributed to the fact that women live longer than men on average, and older age is the greatest risk factor for AD [40].

Living alone with SCD makes this affected populace more vulnerable [41,42]. Besides an increased risk for injury to self or others there are more unmet needs such as mobility, adherence to medication schedule, activities of daily living and coping with financial matters. In addition, they are less likely to use health services and are at a risk for poor health outcomes [42]. Over thirty percent of Delawareans with SCD were observed to be living alone.

Significant disparities in functional limitations were observed among Delaware adults with SCD. Delaware adults with SCD and at least one comorbid chronic condition were more likely to report SCD-related functional limitations (*p* < .05). Nearly 4 in 10 of Delawareans with SCD and a chronic condition reported they had to give up day-to-day activities such as cooking, cleaning, or paying bills and needed assistance because of their memory problems. More than 1 in 3 Delaware adults with comorbid SCD and chronic condition say their worsening memory problems interfere with their ability to work, volunteer, or engage socially. Taken together, nearly 73% of Delaware adults with SCD and at least one chronic condition say it creates “functional difficulties”—that is, their memory problems disrupt everyday tasks and/or interfere with work or social activities. Functional limitations increase demand for formal and/or informal caregiver support and tend to overwhelm available resources [43,44].

Over 68% percent of Delaware adults with SCD (and no comorbid chronic condition) reported not discussing their more frequent of worsening confusion or memory loss with a health care professional. Prior studies have reported up to 54% of those who reported SCD, had not consulted a health care professional [20,22]. Healthy People 2030, a vital framework for prioritizing health issues in U.S. has brought the topic of SCD to the forefront by including a new objective of increasing the proportion of adults with SCD who discuss their confusion or memory loss with a health‐care professional over the next decade [45,46]. This encourages states to make SCD a high -priority issue—engage in targeted interventions- and to collect SCD data on an ongoing basis to measure progress [10,46].

Findings in this study are subject to several limitations. First, BRFSS survey samples are collected from noninstitutionalized adults. People living in long-term care facilities and nursing homes may not be included. SCD and chronic conditions prevalence is bound to be higher among older adults living in these facilities. Second, SCD as defined is self-reported, and not an objective assessment. Third, adults with severe cognitive impairment may be limited in their capacity to participate in the survey. Despite these limitations, the BRFSS SCD module is an all-important indispensable tool to provide population- based data and a viable alternative resource to inform public health policy and decision-making on cognitive health issues [10,18]. Consistency of the association between symptoms of depression and SCD has been well documented [47] and indicates that mood symptoms be considered in those who present with SCD. Since this study did not assess correlation of SCD symptoms with mood disorders, it is duly acknowledged to be a limitation. It is to be noted that subjects included in this study come from two rounds (2016 and 2020), with a 4-year period between measurements. However, this was no ordinary gap-the year 2020 saw the most severe stage of the COVID pandemic. It is quite likely that during 2020 the prevalence of depression and mood disorders increased, thus leading to an increased reporting of cognitive impairment.

Another obvious limitation is a fact that this study did not conduct adjusted analyses and the findings are to be interpreted with caution.

## Conclusion

Delawareans are living longer than ever before—and that is raising a new challenge that can be taken as an opportunity [9,20]. Considering that by 2030 the proportion of Delawareans over 60 will show a 50.4 percent increase [3], the state cannot afford to postpone public policies to address the dual burden of SCD and chronic conditions. Results from this study can help public health stakeholders in Delaware to be better informed and prepared for the challenges associated with cognitive decline and comorbidity. Delaware’s successful initiatives in this regard may well serve as a bellwether, providing a road map for other states.
